# Preclinical efficacy of Sym004, novel anti-EGFR antibody mixture, in esophageal squamous cell carcinoma cell lines

**DOI:** 10.18632/oncotarget.14209

**Published:** 2016-12-26

**Authors:** Shota Fukuoka, Takashi Kojima, Yoshikatsu Koga, Mayumi Yamauchi, Masayuki Komatsu, Rie Komatsuzaki, Hiroki Sasaki, Masahiro Yasunaga, Yasuhiro Matsumura, Toshihiko Doi, Atsushi Ohtsu

**Affiliations:** ^1^ Division of Gastrointestinal Oncology, National Cancer Center Hospital East, Kashiwa, Japan; ^2^ Division of Developmental Therapeutics, Exploratory Oncology Research and Clinical Trial Center, National Cancer Center Hospital East, Kashiwa, Japan; ^3^ Department of Translational Oncology, Fundamental Innovative Oncology Core Center, National Cancer Center Research Institute, Tokyo, Japan; ^4^ Exploratory Oncology Research and Clinical Trial Center, National Cancer Center, Kashiwa, Japan; ^5^ Advanced Clinical Research of Cancer, Juntendo University Graduate School of Medicine, Tokyo, Japan

**Keywords:** esophageal squamous cell carcinoma, epidermal growth factor receptor, Sym004

## Abstract

Epidermal growth factor receptor (EGFR) is a well-validated oncological target molecule for monoclonal antibody therapies and Sym004 is a novel anti-EGFR antibody mixture comprising two recombinant chimeric IgG1 antibodies against non-overlapping epitopes of EGFR. Because EGFR is highly expressed in the majority of esophageal squamous cell carcinomas (ESCCs), we investigated the efficacy of Sym004 in human ESCC cell lines. Forty eight ESCC cell lines were treated with three kinds of anti-EGFR antibodies (Sym004, cetuximab, and panitumumab). Genetic background was investigated by next generation sequencing. The internalization of anti-EGFR antibodies into ESCC cells and inhibition of the EGFR signaling cascade by anti-EGFR antibodies were investigated *in vitro*. Furthermore, growth inhibition by anti-EGFR antibody treatment was investigated *in vitro* and *in vivo*. Sym004 treatments were more effective at inducing EGFR internalization and degradation than the two other anti-EGFR antibodies. Sym004 was more sensitive significantly to cell lines with EGFR gene amplification than those without amplification (*P* = 0.002). Growth inhibition of Sym004 was greater than in that of cetuximab or panitumumab *in vitro* and *in vivo*. These studies showed that Sym004 exhibited antitumor activity in some ESCC cell lines in preclinical settings and warrant a clinical evaluation in patients with ESCC. *EGFR* amplification is a potential biomarker of response to Sym004.

## INTRODUCTION

Esophageal cancer (EC) is the 6th leading cause of cancer death and 8th most common cancer worldwide [1]. Remarkable ethnic differences are seen in EC: more than 95% of EC patients in Asia show squamous cell carcinoma histology. Pathological analyses have shown that high EGFR expression occurs in 70–88% of patients with esophageal squamous cell carcinoma (ESCC) which correlates with poor prognosis [2–6]. Moreover, head and neck squamous cell carcinoma (HNSCC) exhibits high levels of EGFR expression with comparable tumor biology to ESCC. The efficacy of cetuximab, an anti-EGFR antibody, in patients with HNSCC has been demonstrated in combination with radiotherapy or conventional chemotherapy. [7, 8]. Based on these evidences, EGFR-targeted therapy is hypothesized to be effective for the treatment of ESCC, particularly for Asian patients.

Sym004 is a 1:1 mixture of the two novel chimeric IgG1 anti-EGFR monoclonal antibodies (mAb) mAb 992 and mAb 1024. These antibodies bind non-overlapping epitopes on the extracellular domain III of EGFR and the primary mechanism of action of Sym004 is thought to be EGFR cross-linking, internalization and degradation of the EGFR from the cell surface [9]. Although considerable *in vitro* and *in vivo* preclinical evidence suggests that Sym004 is superior to cetuximab and panitumumab in a several types of cancer, its efficacy has not yet been demonstrated in ESCC [9–13].

In this study, we used 48 ESCC cell lines and three kinds of anti-EGFR antibodies (Sym004, cetuximab, and panitumumab) to analyze the efficacy of anti EGFR antibodies both *in vitro* and *in vivo*.

## RESULTS

### Sym004 inhibited growth of ESCC cell lines

**Figure 1:**
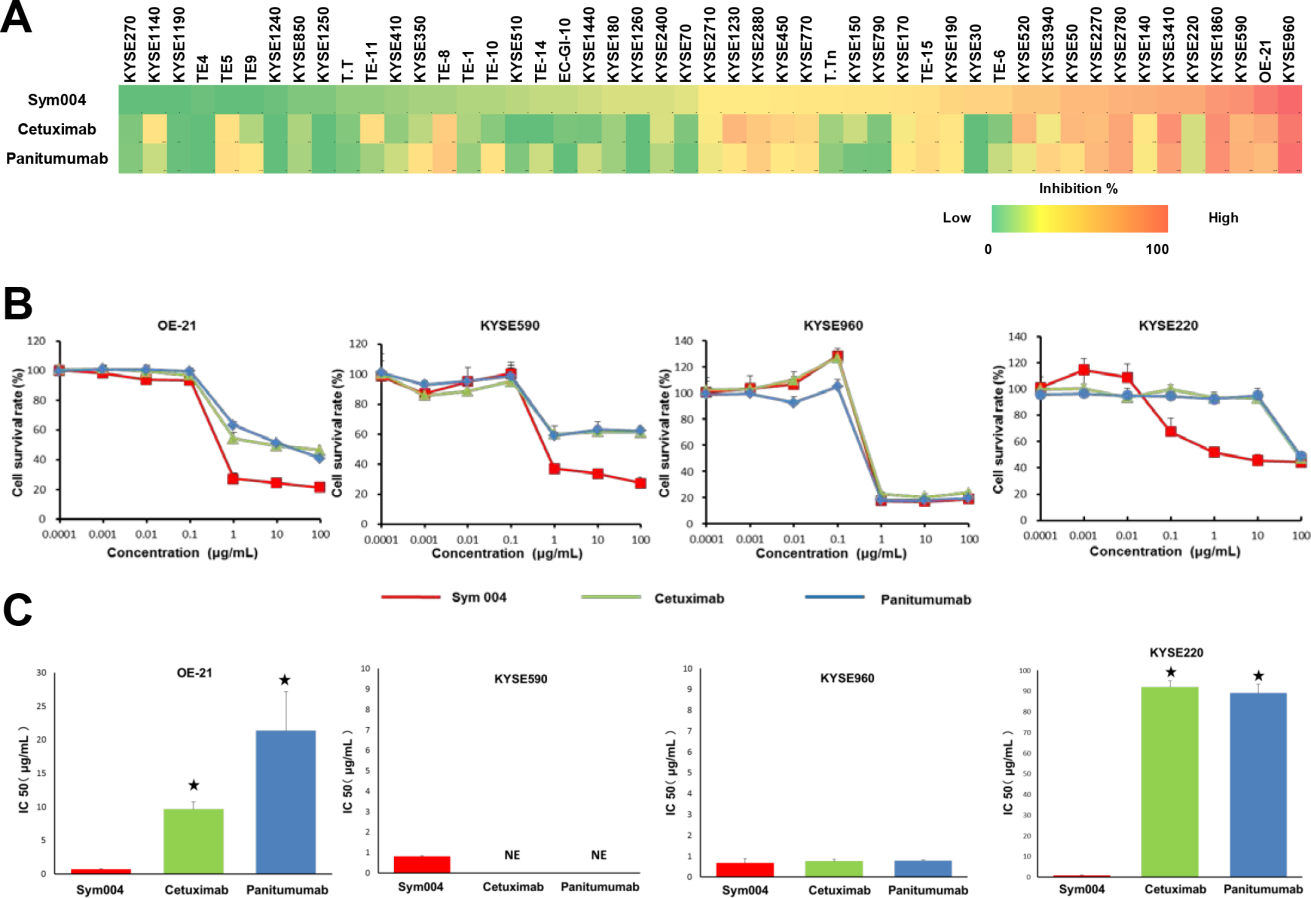
Growth inhibition assay using anti-EGFR antibodies in ESCC cell lines (A) Effect of Sym004, cetuximab and panitumumab on cell proliferation in a panel of 48 ESCC cell lines. Each cell line was treated with 1 μg/mL of each anti-EGFR antibody for 96 h, and the number of viable cells was measured by conventional WST-8 assay according to the manufacturer's instructions. All experiments were repeated three times independently, and cell growth inhibition rate at 1 μg/mL of Sym004, cetuximab, and panitumumab are represented by heat map, respectively (0 to 100 %). (B) Comparisons of cell growth inhibition activity of the three anti-EGFR antibodies. The anti-proliferative activity of Sym004 was greater than that of other anti-EGFR antibodies in OE-21, KYSE590 cells which are EGFR amplified and KYSE220 cells which are non-EGFR amplified. (C) Comparisons of IC 50 of anti-EGFR antibodies. ⋆: P < 0.05, Sym004 vs cetuximab or panitumumab (One way ANOVA with Dunnet's test). NE: no effect. The IC 50 values of Sym004 were significantly lower than those of cetuximab and panitumumab in OE-21, KYSE220 cells.

In the present study, we compared the growth inhibitory effects of Sym004, cetuximab and panitumumab in a panel of 48 ESCC cell lines at 1 μg/mL (Figure 1A). In 34 of the 48 cell lines tested Sym004 showed more potent cytotoxic effect than cetuximab and panitumumab. Seven of 48 cell lines were 50% inhibited by Sym004 at 1 μg/mL, whereas only 3 cell lines were 50% inhibited by cetuximab and panitumumab at this dose. Although the effects of these antibody preparations were similar in KYSE960 cell, the anti-proliferative activity of Sym004 was more potent than those of the two commercially available anti-EGFR antibodies in OE-21, KYSE590, and KYSE220 cells (Figure 1B). The IC 50 values of Sym004 were significantly lower than those of cetuximab and panitumumab in OE-21, KYSE220 cells (Figure 1C).

### Relationship between genetic background and response to Sym004

**Figure 2:**
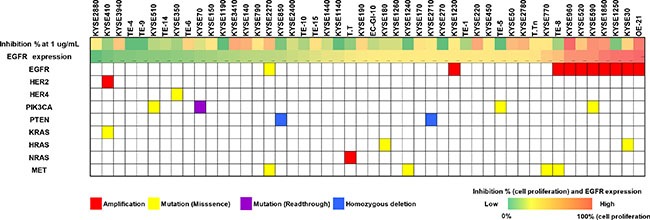
Relationship between genetic background status and Sym004 inhibitory effects of cell in ESCC cell lines Percent inhibition of cell proliferation (0 to 100%) at 1 μg/mL Sym004 and EGFR expression levels by Western blot analysis are represented by heatmap. Mutation statuses and effects on the key components of cancer signaling pathways including loss of tumor suppressors and gene amplifications were determined using NGS. Mutation statuses were indicated as follows: red, amplifications; yellow, mutations (missense); purple, mutations (read-through); and blue, homozygous deletions.

To identify the potential biomarkers responses to Sym004 in ESCC lines, we investigated genetic background in 50 cancer-related genes including loss of tumor suppressor gene and gene amplification, and effects on the key components of cancer-associated signaling pathways (Figure 2). In these experiments, *EGFR* amplification was found in 9 cell lines (18.7%) including OE21, KYSE590, and KYSE960 and mutations of oncogenes *MET*, *PIK3CA*, *KRAS*, and *HRAS* were detected in 8.3%, 8.3%, and 6.3% of cell lines, respectively.

**Figure 3:**
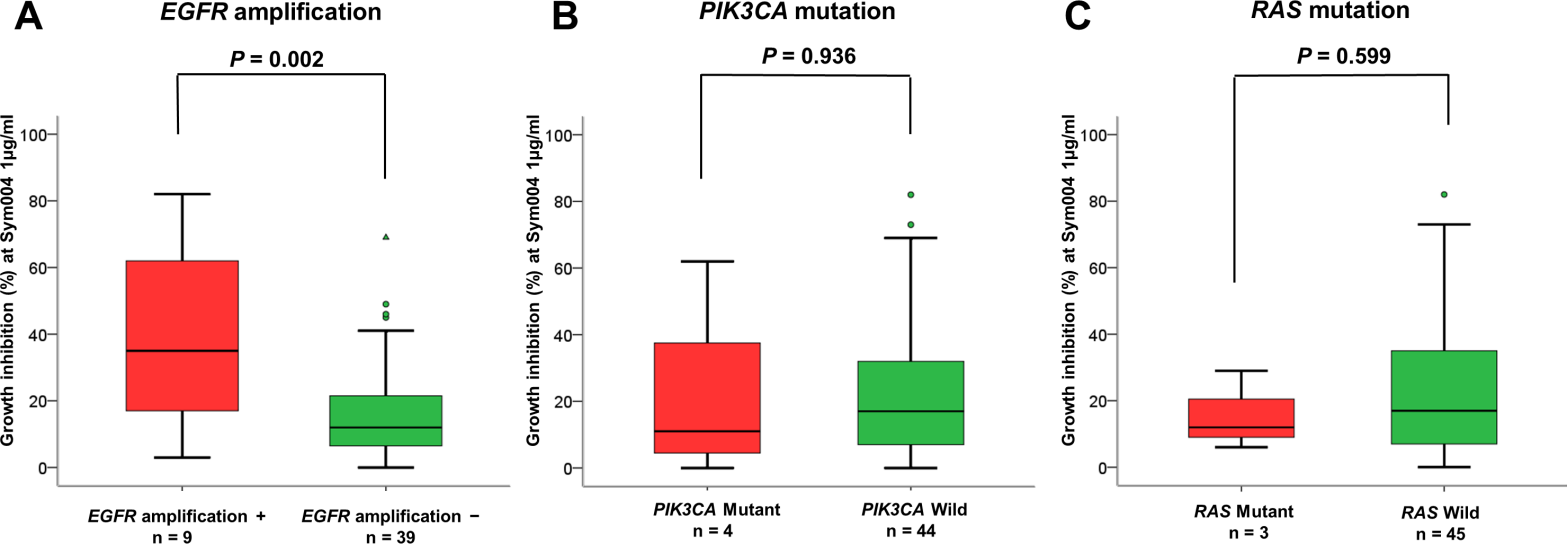
Relationship between Sym004 sensitivity and oncogene activation status Cell lines were classified into groups of EGFR amplification + and −, PIK3CA mutant and wild type, and RAS mutant and wild type. Relationships between growth inhibitory activities of Sym004 and oncogene activation status were analyzed. The percentages of growth inhibition with treatment at 1 μg/mL of Sym004 were plotted as box plot, and medians of the group were labeled on each plot as the black bar. Circles indicate outliers with values between 1.5 and 3 box lengths from the upper or lower edge of the box. Triangles indicate outliers beyond 3 box lengths from the edge of the box. Cell lines with EGFR gene amplification showed significantly higher sensitivity to Sym004 than without amplification (P = 0.002). P values were determined by Student's t-test.

Cell lines with *EGFR* amplification showed significantly greater (*P* = 0.002) sensitivity to Sym004 than those without *EGFR* amplifications (Figure 3A). However, no difference in the sensitivity was observed between cells with mutations in *PIK3CA* and *RAS* and those without mutation (Figure 3B and 3C).

### Internalization of Sym004 into cells

**Figure 4:**
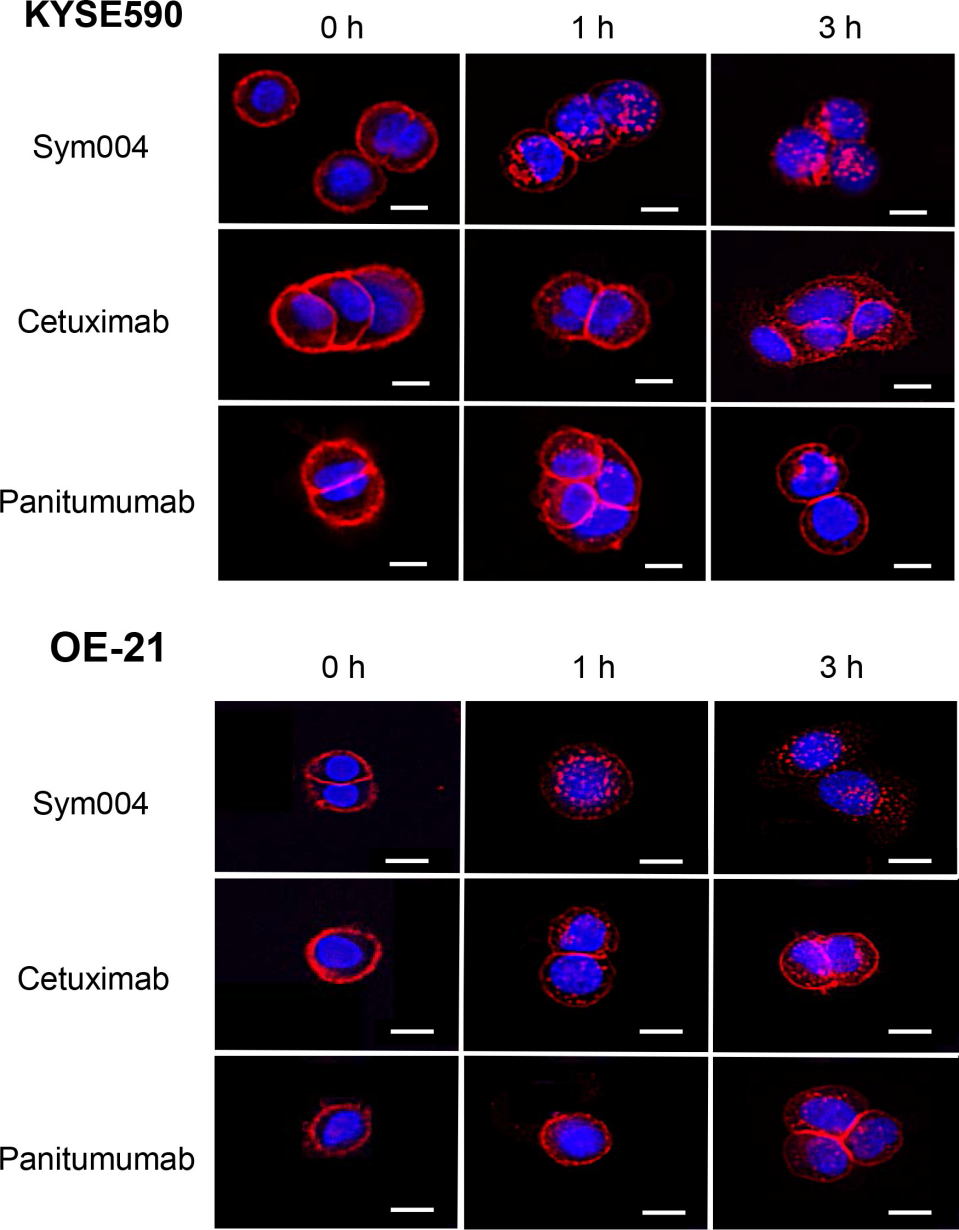
Internalization of Alexa Fluor 647-conjugated anti-EGFR antibodies in KYSE590 cells and OE-21 cells Sym004 was effectively internalized in comparison with cetuximab or panitumumab. Scale bars; 10 μm. Red; anti-EGFR antibodies, blue; the nucleus.

All anti-EGFR antibodies were located in cell surface membrane at 0 h incubation (Figure 4). In almost cell lines tested, Sym004 was sufficiently internalized into the cytoplasm even after 1h incubation. However, most of the cetuximab and panitumumab were still located on the cell surface and cells contained only few visible intracellular vesicles after 1h and even after 3 h in KYSE590 and OE-21 cell lines.

### Degradation of EGFR protein and down regulation of EGFR signaling cascade by Sym004

**Figure 5:**
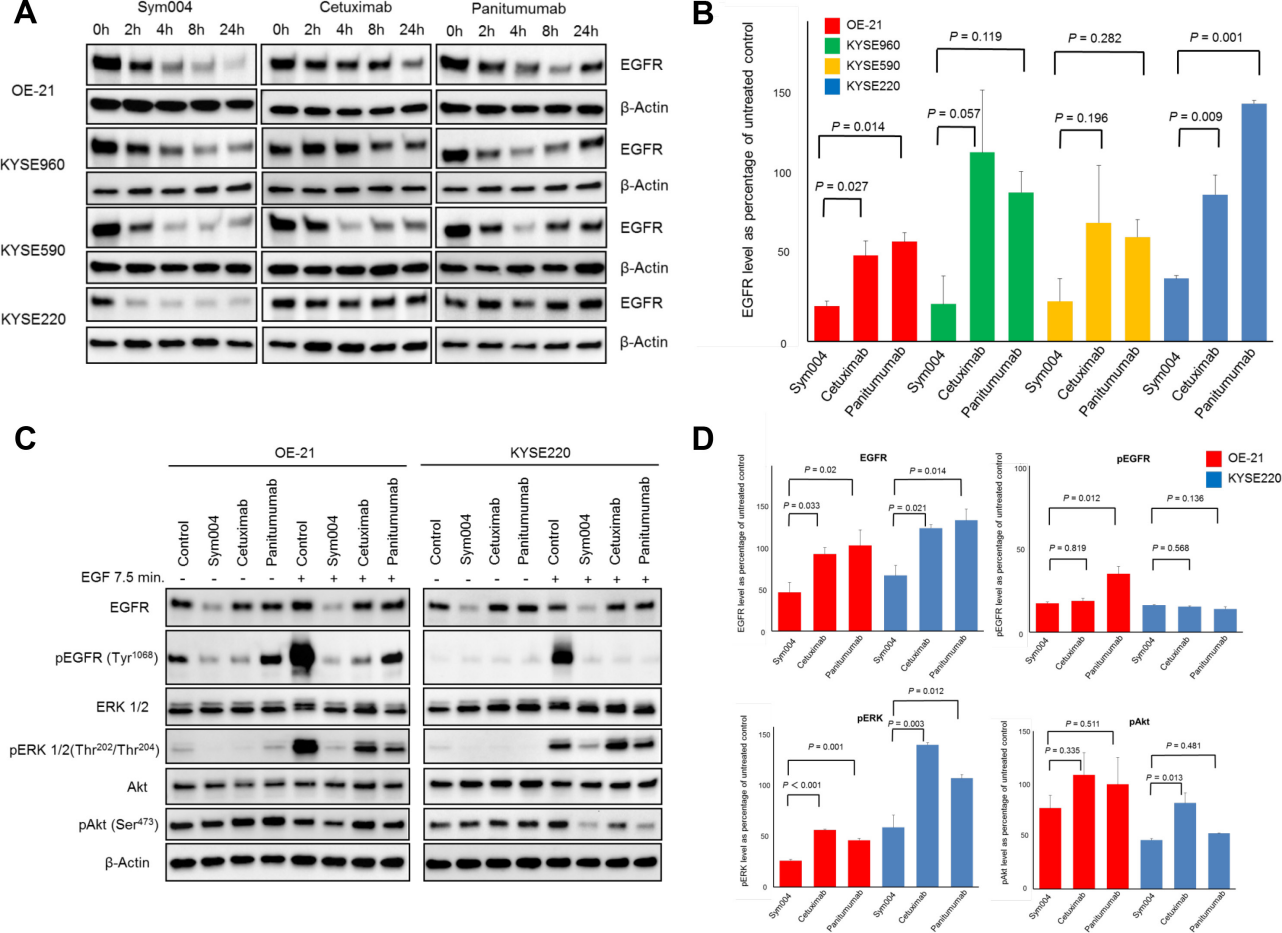
Effects of anti-EGFR antibodies on EGFR protein expression (A) OE-21, KYSE960, KYSE590 and KYSE 220 cell lines were treated with 10 μg/mL of anti-EGFR mAbs for 2, 4, 8, and 24 h. Cells were then lysed and cell extracts were analyzed using Western blotting for EGFR and beta-actin (loading control). (B) Band intensities at 24 h (from Figure 5A) were quantified, and EGFR expression levels were normalized to those of β-Actin. Reduction of EGFR protein was observed in OE-21 and KYSE 220 cells treated by Sym004 significantly more effective than those by cetuximab (P = 0.027 and P = 0.009, respectively) and panitumumab (P = 0.014 and P = 0.001, respectively). (C) The immunoblot analyses of the effect of EGF stimulation on phosphorylation of EGFR and downstream signaling molecules in OE-21 and KYSE220 cell lines following 8 h of treatment of Sym004. (D) Quantification of band intensities of EGFR, pEGFR, pERK and pAkt relative to untreated control in the presence of ligand. In OE-21 and KYSE220 cells, Sym004 treatment led to a significantly more potent blockade phosphorylation of ERK than cetuximab and panitumumab in the presence of ligand.

EGFR protein of OE-21, KYSE960, KYSE590 and KYSE220 cells treated with 10 μg/mL of each antibody for 2, 4, 8, or 24 h were investigated by Western blotting analysis. EGFR levels were dramatically decreased by Sym004 in all three cell lines, whereas small decrease in EGFR level was observed by cetuximab or panitumumab (Figure 5A). Quantification of band intensities showed that Sym004 reduced the total EGFR level by 60 to 80% within 24 h in the four cell lines (Figure 5B). In OE21 cells and KYSE220, reduction of EGFR protein by Sym004 was significantly more effective than cetuximab (*P* = 0.027 and *P* = 0.009, respectively) and panitumumab (*P* = 0.014 and *P* = 0.001, respectively). To clarify the mechanisms underlying the superior inhibitory effects of a Sym004 in the presence of ligand, the phosphorylation of EGFR and the status of downstream signaling molecules was investigated in OE-21 and KYSE220 cell lines (Figure 5C). In the presence and absence of ligand, Sym004 treatment led to a more potent blockade of EGFR phosphorylation at the Tyr1068 compared with panitumumab (*P* = 0.012) in OE-21 cells (Figure 5D). In OE-21 and KYSE220 cells, similar results were found for phosphorylation of ERK in the presence of ligand. Sym004 was also more potent than cetuximab at inhibiting phosphorylation of AKT in the KYSE220 cell line.

### Tumor growth inhibition by anti-EGFR antibodies *in vivo*

**Figure 6:**
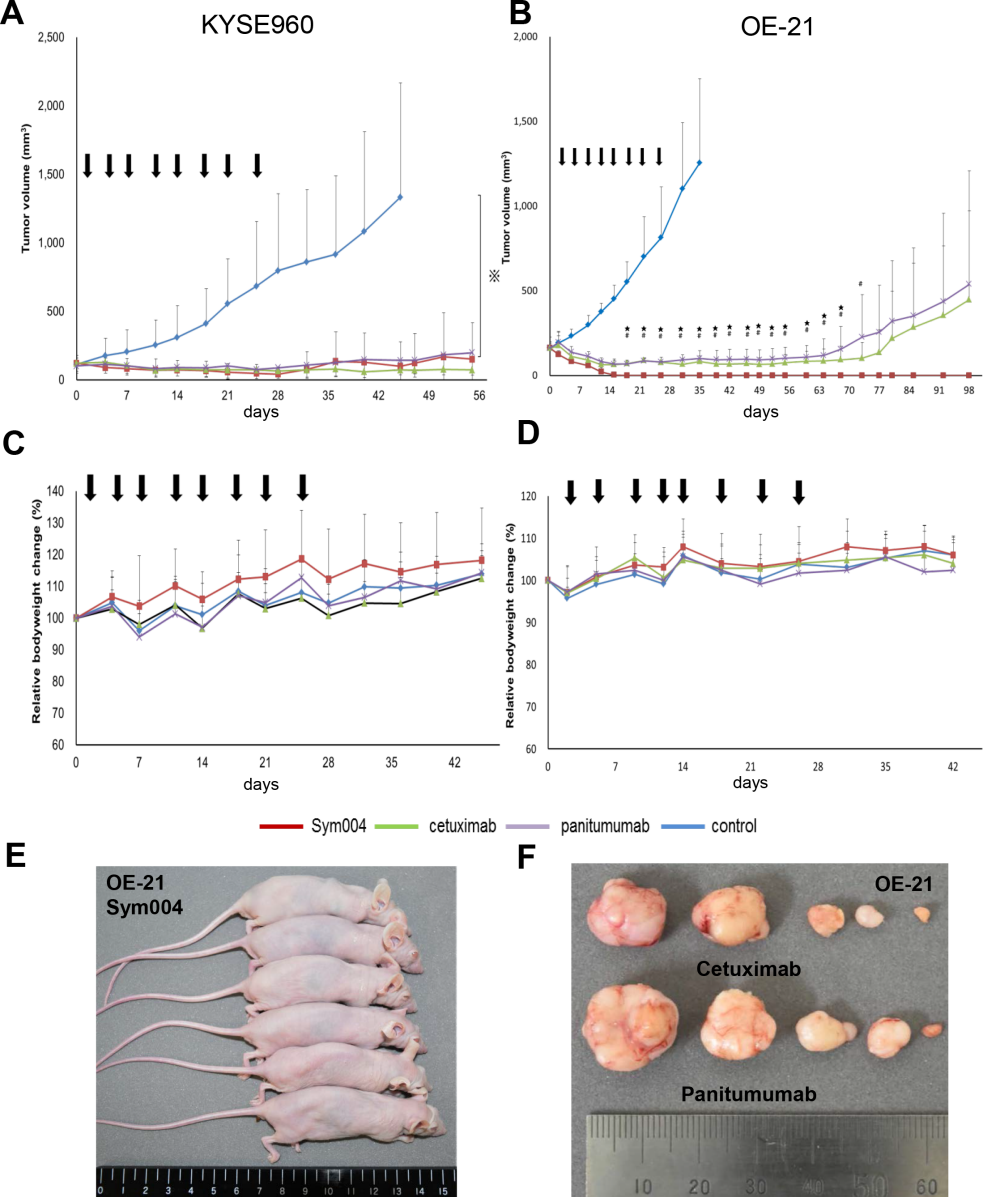
Anti-tumor effect of anti-EGFR antibodies in KYSE960 and OE-21 xenograft models Points, mean; bars, SD; arrows, drug injections. ⋇:P < 0.001, Control vs anti-EGFR antibodies (One way ANOVA with Tukey test). #:P < 0.05, Sym004 vs panitumumab, ⋆:P < 0.05, Sym004 vs cetuximab (One way ANOVA with Dunnet's test). (A) Anti-tumor effects of anti-EGFR antibodies in KYSE960 xenograft model. Mice bearing KYSE960 xenografts were treated with Sym004 (50 mg/kg), cetuximab (50 mg/kg), and panitumumab (50 mg/kg) for 28 days (N = 6 in each group). Tumor growth was significantly suppressed by all anti-EGFR antibodies compared to saline (P < 0.001). (C) Comparable anti-tumor effects of anti-EGFR treatment were observed without negative effects on body weight gain. (B) Anti-tumor effects of anti-EGFR antibodies in OE-21 xenograft model. Mice bearing OE21 xenografts were treated with Sym004 (50 mg/kg), cetuximab (50 mg/kg), and panitumumab (50 mg/kg) for 28 days (N = 6 in each group). Although all anti-EGFR antibodies induced remarkable tumor regression in the OE21 model, Sym004 induced the most potent effect on both complete regression and tumor regression levels. (D) No difference of effect on body weight among the tested anti-EGFR antibodies was observed. (E) Photograph of OE-21 xenograft tumor mice treated by Sym004 at 98 days. (F) Photograph of the harvested tumors treated by cetuximab and panitumumab at 98 days.

The tumor growth of KYSE960 cells was significantly suppressed by all groups treated with anti-EGFR antibodies compared with the group treated with saline (*P* < 0.001) (Figure 6A). Body weight loss was not observed in any groups (Figure 6C). Subsequently, xenograft tumors were established from OE-21 cells, and the effects of the three anti-EGFR antibody preparations were compared following i.p. injections (Figure 6B). In this OE-21 xenograft model, 50 mg/kg Sym004 caused rapid and complete response of all tumors and maintained no tumor recurrence for over 90 days after treatment (Figure 6E). In the cetuximab group (1/6) and panitumumab group (1/6), complete response was also observed. However, the other of them were regrowth during the observation period (Figure 6F). Importantly, no differences in body weight changes as an adverse effect were observed between anti-EGFR antibody treatment groups (Figure 6D).

## DISCUSSION

Overexpression of the EGFR is correlated with prognosis of several kind of cancer and activated EGFR signals via the RAS, ERK1/2 and PI3K/Akt pathways. This correlation may result in chemotherapeutic resistance, angiogenesis, and enhanced tumor progression [14]. Hence, EGFR-targeted therapy is a promising approach for cancer treatment and significant efficacy of anti-EGFR antibody, such as cetuximab, has been shown in HNSCC. However, in recent clinical trials of ESCC, combination treatment with cetuximab and radiotherapy or conventional chemotherapy failed to show significant additional treatment efficacy [15–17]. Previous studies have reported that mixtures of antibodies targeting multiple distinct epitopes are more effective than single mAbs [18]. Sym004 was developed following screening of more than 400 different mAb combinations based on the highest capacity to inhibit cell growth. Sym004 is a mixture of anti-EGFR mAb 992 and 1024 that targets non-overlapping epitopes (epitope 992 vs. 1024) in EGFR extracellular domain III. These two epitopes are different from the epitopes of cetuximab and panitumumab [11, 19]. Therefore, Sym004 is hypothesized to be more effective than approved anti-EGFR monoclonal antibodies for the treatment of ESCC.

In the present study, Sym004 exerted more potent growth inhibition than cetuximab or panitumumab in a panel of 48 ESCC cell lines. Interestingly, ESCC cell lines with *EGFR* amplification tended to be more sensitive to Sym004 in *in vitro* cell proliferation assays (Figure 3A). Cetuximab was initially approved for patients with EGFR overexpressing colorectal cancer. However, following retrospective studies failed to show correlation between efficacy of the agent and intensity of EGFR-overexpression [20, 21], although there was positive correlations between *EGFR* amplification and responses to anti-EGFR monoclonal antibody–based treatments [22, 23].

The clinicopathological analysis of ESCC showed that *EGFR* amplification occurred in 24%–28% of ESCC patients and were significantly associated with high-level overexpression of EGFR [24, 25]. In our study, *EGFR* amplification was observed more frequently in EGFR relative high expression cell lines (Figure 2). High *EGFR* gene copy number or *EGFR* amplification is correlated with advanced pathologic stage and more number of the metastatic lymph nodes in ESCC [26, 27]. *EGFR* amplification may be helpful in predicting patient's outcome, however, it is not definite as a poor prognosis factor. Although further studies are required to characterize the mechanisms underlying the relationship between *EGFR* amplification and sensitivity to Sym004, the present study showed that the importance of the internalization ability of Sym004 following the binding of the mAb to EGFR suggesting that high level presentation of EGFR on cancer cells is associated with responses to Sym004.

*KRAS* mutations in the exon 2 are reportedly predictive of resistance to anti-EGFR mAb therapy, and a retrospective analysis of *RAS* mutations in specimens from a randomized trial of combination chemotherapy for metastatic colorectal cancer (the PRIME trial) indicated that, similar to *KRAS* exon 2, mutations in *KRAS* exons 3 and 4 and *NRAS* exons 2, 3, and 4 were negative predictive factors to panitumumab [28]. However, *KRAS* and *NRAS* mutations are rarely observed in esophageal cancer [25]. In HNSCC, *HRAS* mutations are more common than *KRAS* mutations [29]and this mutation exhibited *de novo* resistance to cetuximab-based therapy [30]. In our study, *KRAS* and *HRAS* mutations were found in only one and two of 48 ESCC cell lines, respectively, indicating no significant correlation between Sym004 responses and these mutations. Similarly, relationships between *PIK3CA* mutations and responses to anti-EGFR mAb remain controversial [22, 23, 31], and the present data showed no differences in Sym004 sensitivity between *PIK3CA* mutant and wild-type cancer cells.

The lack of statistically significant difference between Sym004 and the cetuximab or panitumumab in the KYSE960 xenograft model was not surprising, because *in vitro* result showeds that Sym004 and the other anti-EGFR mAb were equally effective in the growth of KYSE960 cells. On the other hand, tumor eradication of OE-21 xenografts was achieved in all of the mice treated with Sym004 without any recurrences during the observation period. Sym004 was significantly more potent in tumor growth inhibition than cetuximab or panitumumab, respectively. This difference of anti-tumor effect in OE-21 cell line can be explained by the enhanced capability of Sym004 to induce EGFR internalization and degradation.

## MATERIALS AND METHODS

### Cell culture

TE series and EC-GI-10 cell lines were established from Japanese patients and were obtained from Japanese Collection of Research Bioresources (Tsukuba, Japan). KYSE series cell lines established from Japanese patients were obtained from the Health Science Research Resources Bank (Osaka, Japan). OE-21 established from British was obtained from the European Cell Culture Society (Wiltshire, UK). Cells were maintained in appropriate medium supplemented with 10% fetal bovine serum (Cell Culture Technologies, Gaggenau-Hoerden, Germany), 100-units/mL penicillin, 100 μg/mL streptomycin and 25 μg/mL amphotericin B (Sigma, St.Louis, MO) in a humidified atmosphere containing 5% CO2 at 37°C.

### Compounds and antibodies

Sym004 was provided by Merck Serono Co., Ltd. (Tokyo, Japan). Commercially available cetuximab (Erbitux; Merck Serono Co., Ltd) and panitumumab (Vectibix; Amgen, Tokyo, Japan) were used in this study. Antibodies against EGFR (D38B1), phosphorylated EGFR (Tyr^1068^), Akt, phosphorylated-Akt (Ser^473^), ERK1/2, phosphorylated-ERK1/2 (Thr^202^/Thr^204^) and β-actin were purchased from Cell Signaling Technology (Beverly, MA).

### Western blotting analysis

Cells were lysed in a radio immunoprecipitation assay (RIPA) buffer containing 50 mM Tris-HCl (pH8.0), 150-mM sodium chloride, 0.5% sodium deoxycholate, 0.1% sodium dodecyl sulfate, and 1% NP-40 (Wako, Osaka, Japan) with protease inhibitor and phosphatase inhibitor (Wako). Cell extracts were denatured, subjected to sodium dodecyl sulfate polyacrylamide gel electrophoresis (SDS-PAGE), and transferred to polyvinylidene difluoride (PVDF) membranes (Bio-Rad, Hercules, CA) using a Trans-Blot Turbo transfer machine (Bio-Rad). Membranes were then placed in a protein detection system (SNAP i.d.; Millipore, Billerica, MA) and were blocked with phosphate-buffered saline (PBS) containing 0.1% Tween 20 (PBS-T, Sigma) and 0.2% Difco skim milk (Becton Dickinson, Franklin Lakes, NJ). Membranes were then incubated in dilution buffer (0.3% skim milk in 0.1% PBS-T) with primary antibodies for 10 min at room temperature. Antibodies were used at the following dilutions: against EGF Receptor (1:200), phosphorylated EGFR (1:200), Akt (1:200), phosphorylated-Akt (1:400), ERK1/2 (1:200) and phosphorylated-ERK1/2 (1:400) as indicated. Subsequently, membranes were washed with a tris buffered saline containing 0.1% Tween 20 (TBS-T), and proteins were visualized using ECL prime substrate (GE Healthcare, Piscataway, NJ). For analysis of EGFR signaling, cancer cells were serum starved overnight and pretreated with 10 μg/mL of control mAb, Sym004, cetuximab or panitumumab for 8 h and then left unstimulated or stimulated with 1 nmol/L EGF (R&D Systems) for 7.5 min. Western blots were imaged using a ChemiDoc XRSþ System (Bio-Rad).

### Analysis of genetic background

DNA was extracted from cell lines by using the QIAamp DNA Mini Kit (QIAGEN, Tokyo, Japan) in accordance with manufacturer's instructions and was stored at –20°C until use.

Genetic background were analyzed by an Ion PGM (Life Technologies, Foster City, CA) using an Ion AmpliSeq™ Cancer Hotspot Panel v2, which allows the characterization of mutational hotspots in 50 cancer-related genes according to the manufacturer's instructions.

### Viability assays

The growth-inhibitory effects of Sym004, cetuximab and panitumumab were examined using tetrazolium salt based proliferation assay (WST-8 assay: Dojindo, Kumamoto, Japan). All cell lines were seeded into 96-well plates in sextuplicate at a density of 1.5 × 10^3^ cells/ 100 μL and were incubated for 24 h at 37°C. The cells were then treated with various concentrations of Sym004, cetuximab and panitumumab for a total of 96 h under the same conditions. After the removal of medium, WST-8 solution (10 μL) and medium (90 μL) were added to the wells, and the plates were incubated for a further 2 h at 37°C. Finally, growth-inhibitory effects were assessed using a 96-well spectrophotometric plate reader (SpectraMax 190, Molecular Devices Corp., CA). The 50% inhibitory concentration (IC50) was determined from dose–response curves. All experiments were repeated three times independently.

### Immunofluorescence assays

In order to monitor internalization of the antibodies, they were labeled with Alexa Fluor 647 using antibody labeling kits (Invitrogen, Carlsbad, CA). In these experiments, 1.5 × 10^3^ cells were pre-cultured in culture slides (Corning Incorporated, Corning, NY) and incubated with 20 μg/mL of Alexa647-conjugated antibodies at 37°C for 0, 1 and 3 h. After rinsing with PBS, cells were fixed with 4% paraformaldehyde (Wako) for 10 min and then nuclear stained with DAPI solution (Roche, Basel, Switzerland). Fluorescent images were acquired using a BZ-900 instrument (Keyence, Osaka, Japan).

### Xenograft studies

Cells (approximately 1 × 10^7^ cells) were suspended in 0.2 mL of PBS and were inoculated subcutaneously into the right flanks of 5-week-old BALB/c nu/nu mice (Charles River Laboratories Japan, Yokohama, Japan). Mice were maintained in cages under specific pathogen-free conditions, provided with standard food, and given free access to sterilized water. Mice were monitored daily, and tumor volume was measured one or two times weekly using calipers. Tumor volumes were calculated using the following formula: 1/2 × L × W^2^ (L = length; W = width). When tumor volume was reached to 100 mm^3^, mice were randomly divided into 4 tests group consisting of saline, Sym004, cetuximab and panitumumab group. Antibodies were administered at 50 mg/kg twice weekly by intraperitoneal injection. All animal procedures were carried out in compliance with the Guidelines for the Care and Use of Experimental Animals established by the Committee for Animal Experimentation from the National Cancer Center, Japan. These guidelines meet the ethical standards required by law and also comply with the guidelines for the use of experimental animals in Japan.

### Statistical analysis

All results are expressed as mean ± standard deviation (SD), and statistical significance was analyzed using ANOVA with Tukey or Dunnet multiple comparison, as appropriate. Statistical analyses were performed using IBM^®^ SPSS^®^ Statistics version 21 (IBM Corporation, Armonk, NY). All tests were two-sided, and *P* < 0.05 was considered statistically significant.

## CONCLUSIONS

Sym004 exhibited significant tumor growth inhibition in a subset of ESCC cell lines *in vitro* and *in vivo* with pronounced activities in comparison with other anti-EGFR monoclonal antibodies, cetuximab and panitumumab. These analyses suggest that *EGFR* amplification may be a potential predictive biomarker to Sym004 in clinical implication to ESCC.
